# Supplementary material for: Enhanced specificity of clinical high-sensitivity tumor mutation profiling in cell-free DNA via paired normal sequencing using MSK-ACCESS
Source: Nat Commun. 2021 Jun 18;12:3770. doi: 10.1038/s41467-021-24109-5 (PMC8213710; doi:10.1038/s41467-021-24109-5)
Supplement: Supplementary file 1 — Supplementary information [file 41467_2021_24109_MOESM1_ESM.pdf]

Supplementary Information

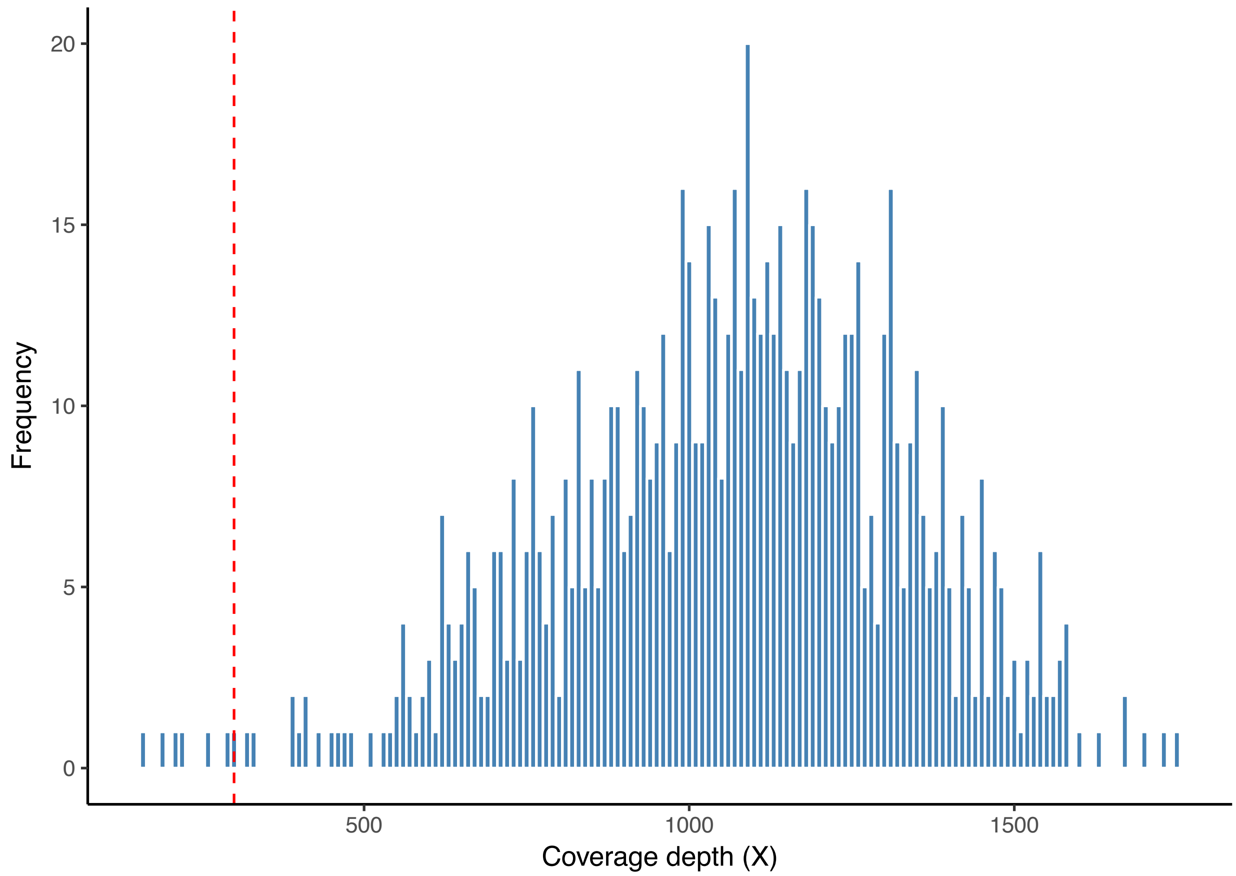

Supplementary Figure 1: MSK-ACCESS exon coverage. Mean coverage of all MSK-ACCESS exons across 47 healthy donor plasma samples.

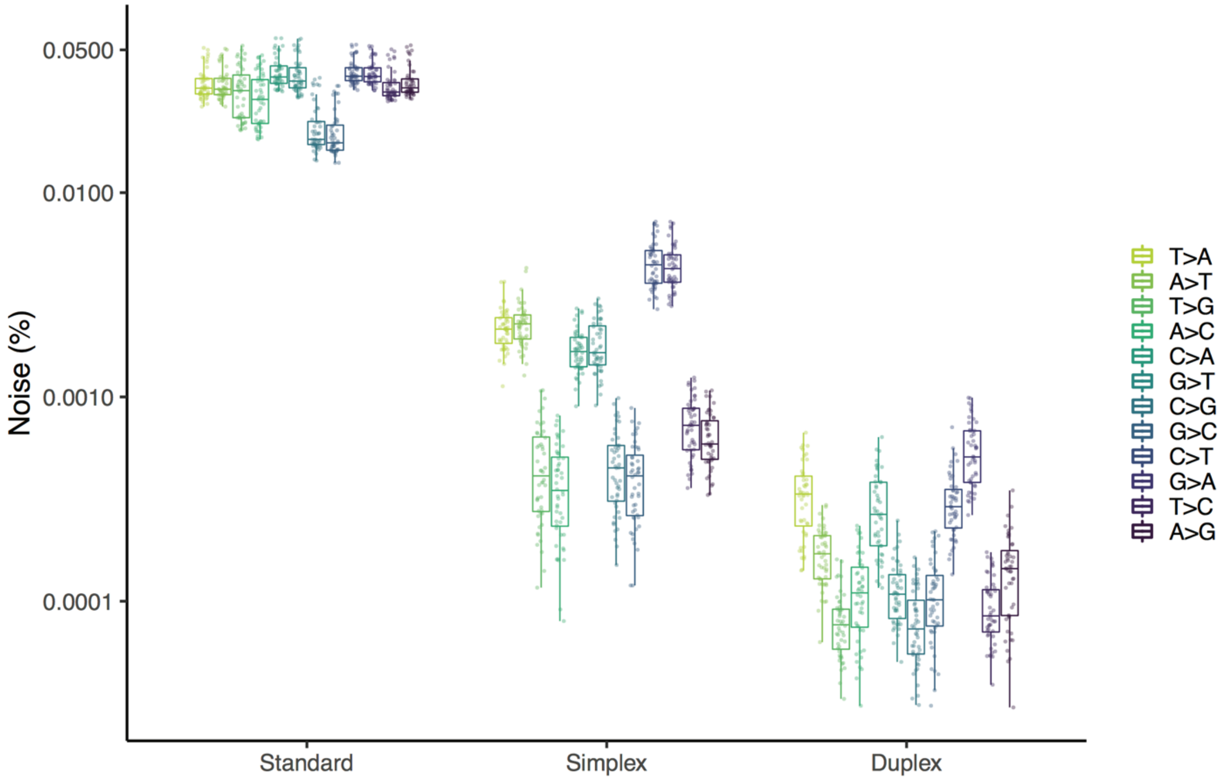

Supplementary Figure 2: HiSeq 2500 error rate. Error rate at sites with non-reference alleles in MSK-ACCESS panel in 47 healthy donor plasma samples sequenced on the HiSeq 2500. All boxplots show the median (center line) and 25th and 75th percentiles (bounding box) along with the 1.5 interquartile range (whiskers).

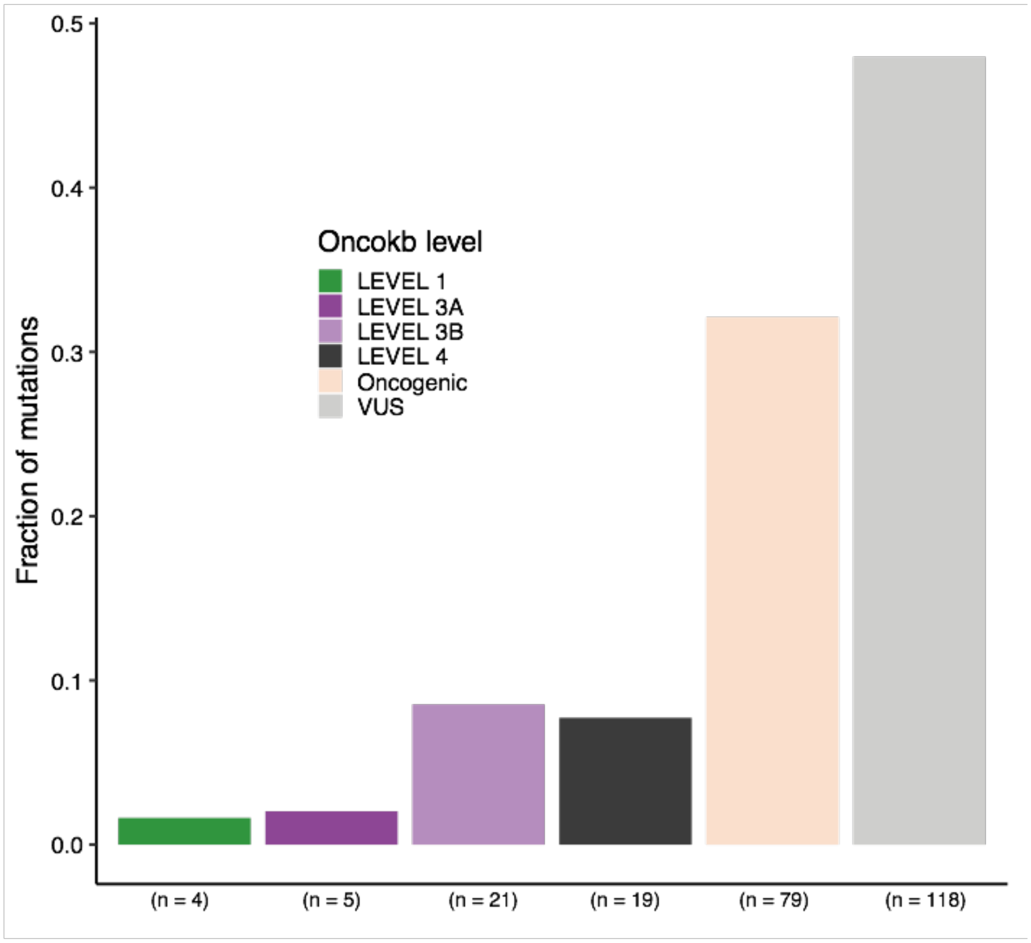

Supplementary Figure 3: OncoKB levels of MSK-ACCESS-only mutations. Fraction of mutations called only by MSK-ACCESS and colored by OncoKB designation

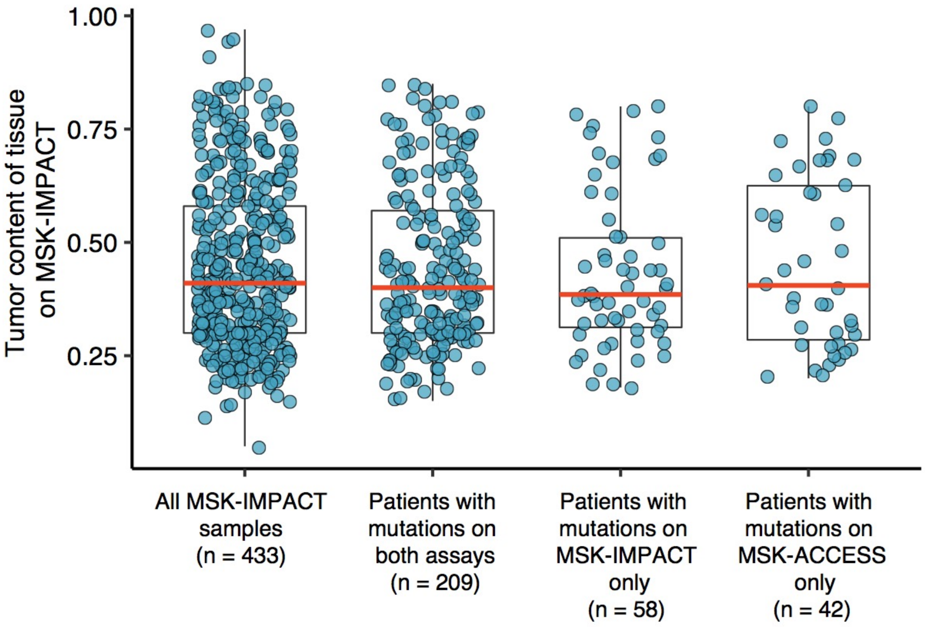

Supplementary Figure 4: Tumor content of MSK-IMPACT tissue samples for patients with actionable mutations (Levels 1-4) used in the concordance analysis. Tumor content of tissue samples were evaluated by FACETS and plotted for i.) all samples, ii.) samples belonging to patients presenting actionable mutations on both assays, iii.) samples belonging to patients with actionable mutations detected in MSK-IMPACT only, and iv) samples belonging to patients with actionable mutations detected in MSK-ACCESS only. All boxplots show the median (center line) and 25th and 75th percentiles (bounding box) along with the 1.5 interquartile range (whiskers).

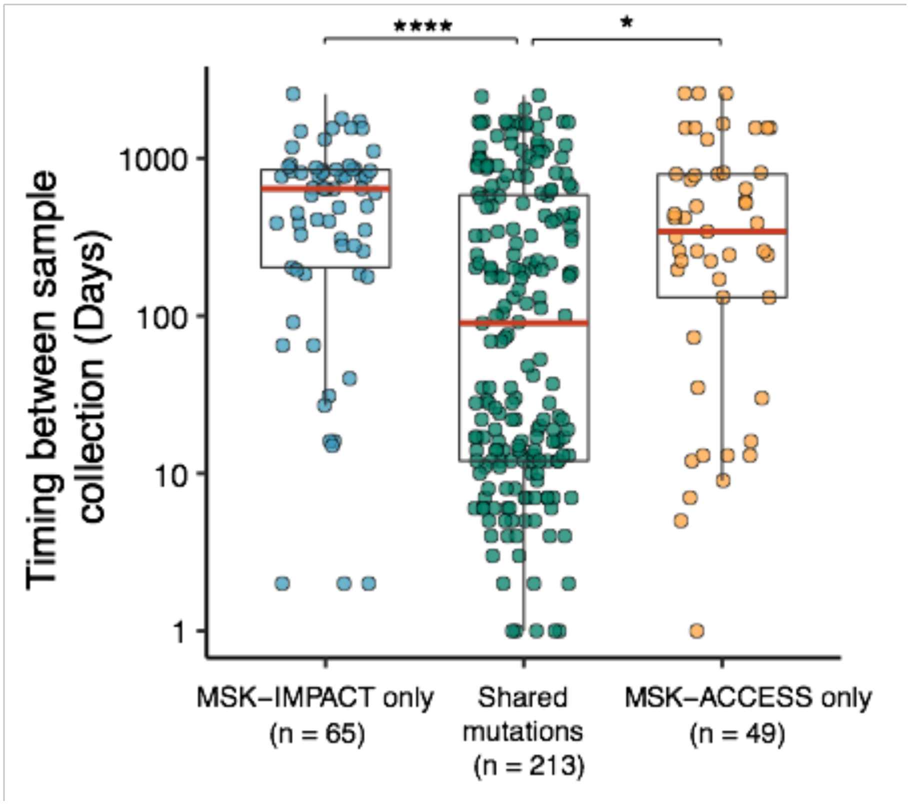

Supplementary Figure 5: Absolute time difference (ΔDOP) between MSK-IMPACT tissue sample and MSK-ACCESS blood sample collection. ΔDOP was evaluated for patients with actionable mutations (Levels 1-4) in MSK-IMPACT only, in both assays, and in MSK-ACCESS only. The p values were obtained from pairwise comparisons using two-sided Mann-Whitney U-tests and adjusted for multiple testing using the Bonferroni method. Patients with actionable mutations only detected on either MSK-IMPACT ($p = 1.94 \times 10^{-5}$) or MSK-ACCESS ($p = 0.0150$) showed a higher ΔDOP than patients for whom actionable mutations were detected on both assays. All boxplots show the median (center line) and 25th and 75th percentiles (bounding box) along with the 1.5 interquartile range (whiskers).

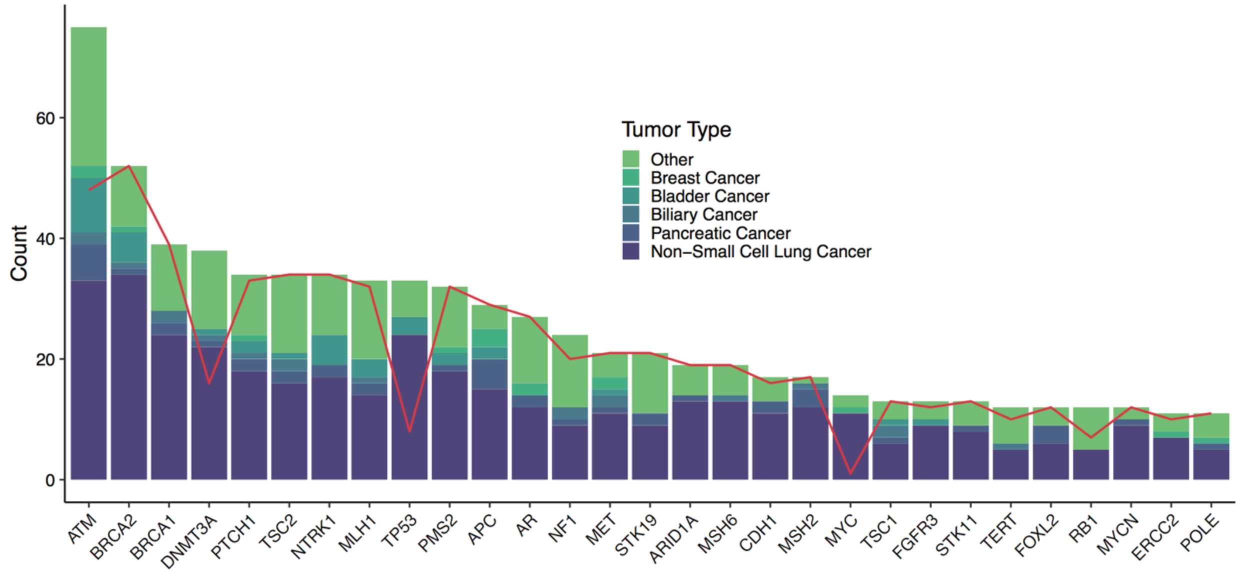

Supplementary Figure 6: Genes filtered out with matched WBC. Number of mutations per gene and tumor type that would remain in clinical samples after filtering with curated healthy normal plasma samples and gnomAD if WBC normal was not used. The red line denotes the number of total mutations that would be present if calls were further filtered for presence in COSMIC hematologic samples; while potential CH mutations would be removed, some tumor derived mutations would also be filtered out.

Patient plasma vs healthy normal plasma sample

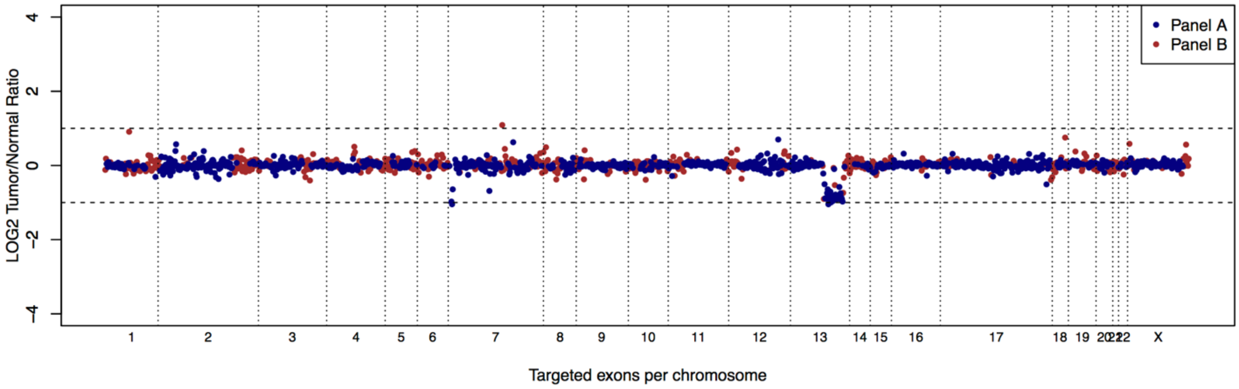

Patient WBC vs healthy normal WBC sample

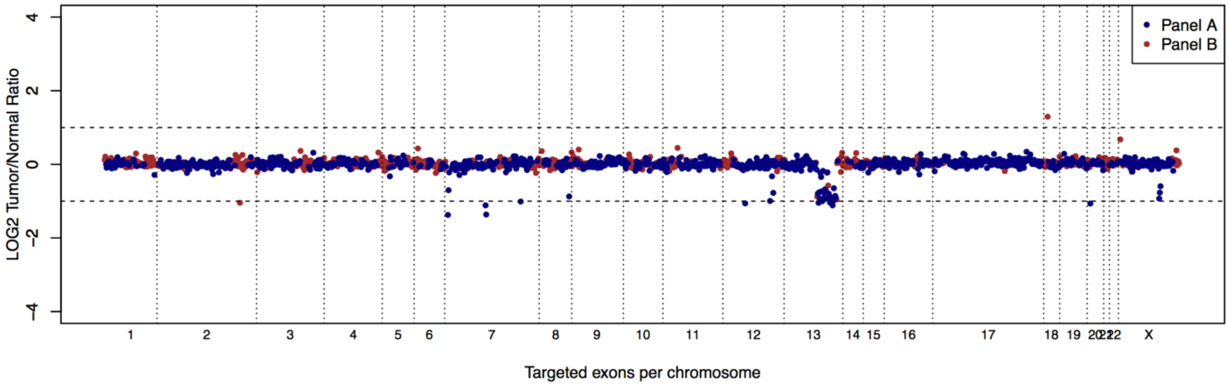

Supplementary Figure 7: Germline copy number deletions in MSK-ACCESS. In both the retinoblastoma cancer patient's plasma and WBC, an *RB1* deletion was detected. Because WBC was sequenced, this deletion was not reported as somatic.

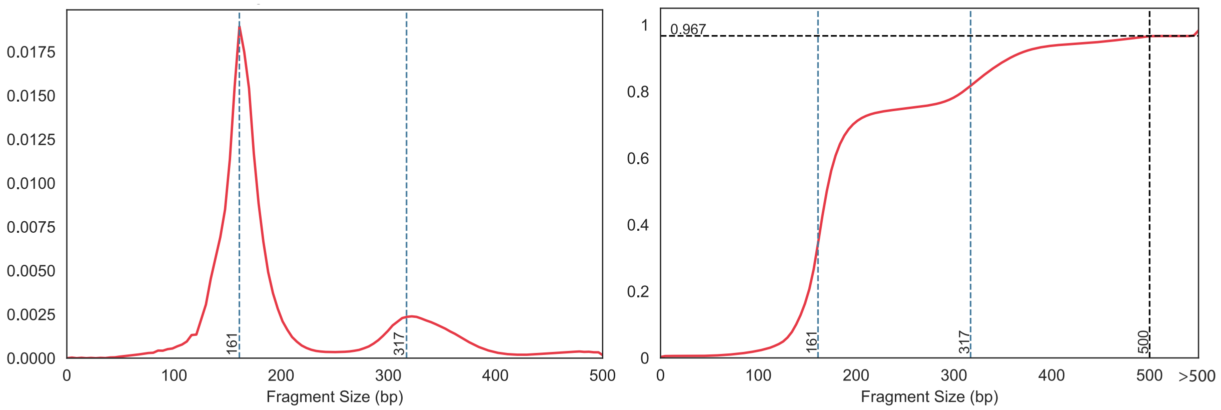

Supplementary Figure 8. Fragment size distribution in plasma samples (a) Probability density function plot indicating an expected bimodal distribution of fragment size with peaks at 161 bp and 317 bp. (b) Cumulative density function plot of the fragment size showing that fragments of sizes 500 bp or lower account for 96.7% of all fragments.

Orthogonal VAF range	MSK-ACCESS called	MSK-ACCESS not called	Detection Type
**0 - 0.01**	21	5	*Genotyping*
**0.01 - 0.05**	35	1	*Genotyping*
**0.05 - 0.1**	10	0	*Genotyping*
**0.1 - 0.15**	6	0	*Genotyping*
**0.15 - 0.2**	10	0	*Genotyping*
**0.2 - 0.3**	5	0	*Genotyping*
**0.3 - 0.4**	3	0	*Genotyping*
**0.4 - 0.5**	1	0	*Genotyping*
**0.5 - 0.6**	1	0	*Genotyping*
**0.6 - 0.7**	1	0	*Genotyping*
**0.7 - 0.8**	1	0	*Genotyping*
**0 - 0.01**	11	15	*De-novo calling*
**0.01 - 0.05**	33	3	*De-novo calling*
**0.05 - 0.1**	10	0	*De-novo calling*
**0.1 - 0.15**	6	0	*De-novo calling*
**0.15 - 0.2**	10	0	*De-novo calling*
**0.2 - 0.3**	5	0	*De-novo calling*
**0.3 - 0.4**	3	0	*De-novo calling*
**0.4 - 0.5**	1	0	*De-novo calling*
**0.5 - 0.6**	1	0	*De-novo calling*
**0.6 - 0.7**	1	0	*De-novo calling*
**0.7 - 0.8**	1	0	*De-novo calling*

Supplementary Table 1. Detection methods for MSK-ACCESS mutations across different ranges of orthogonal VAFs.

Mutation_category	DOP_bin	Mutations	Median_IMPACT VAF	Range_IMPACT VAF	Median_ACCESS VAF	Range_ACCESS VAF
MSK-ACCESS only	<=d3	9	-	-	0.00368	0.00168-0.05407
MSK-ACCESS only	d4-7	9	-	-	0.01604	0.00536-0.02887
MSK-ACCESS only	w1-5	52	-	-	0.00566	0.0005-0.76665
MSK-ACCESS only	w5-10	7	-	-	0.01183	0.0024-0.03745
MSK-ACCESS only	w10>	169	-	-	0.00624	0.00017-0.61606
Shared mutations	<=d3	24	0.2809	0.07556-0.74194	0.01667	0.00095-0.31773
Shared mutations	d4-7	87	0.2665	0.0503-0.91808	0.0589	0.00249-0.55516
Shared mutations	w1-5	202	0.27953	0.02412-0.84899	0.02981	0.00037-0.67852
Shared mutations	w5-10	35	0.28354	0.02593-0.89286	0.03329	0.00041-0.4897
Shared mutations	w10>	358	0.30281	0.02792-0.95931	0.01811	0.00023-0.98964
MSK-IMPACT only	<=d3	7	0.12976	0.07003-0.2551	-	-
MSK-IMPACT only	d4-7	8	0.10417	0.05722-0.37465	-	-
MSK-IMPACT only	w1-5	45	0.25761	0.05488-0.75546	-	-
MSK-IMPACT only	w5-10	6	0.24502	0.02333-0.7861	-	-
MSK-IMPACT only	w10>	188	0.1442	0.01703-0.82803	-	-

Supplementary Table 2: Differences in date of procedure (ΔDOP) for tissue and blood collection and corresponding mutation concordance between MSK-IMPACT and MSK-ACCESS.

<=d3: Less than or equal to 3 days ; **d4-7**: 4 to 7 days ; **w1-5**: 1 to 5 weeks ; **w5-10**: 5 to 10 weeks ; **w10>**: 10 or more weeks

Sample	Chromosome	Position	Ref	Alt	Gene	cDNAchange	AAchange	DP	AD	VAF
P-0024025-T03-XS1	7	55242468	ATTAAGAGAAGCAACATCT	A	EGFR	c.2239_2256delTTAAGAGAAGCAACATCT	p.L747_S752del	1942	1085	0.5587
P-0045809-T01-XS1	7	55259515	T	G	EGFR	c.2573T>G	p.L858R	4745	1863	0.3926
P-0046477-T01-XS1	7	55242464	AGGAATTAAGAGAAGC	A	EGFR	c.2235_2249del	p.E746_A750del	1784	1124	0.63
P-0047033-T01-XS1	3	178936082	G	A	PIK3CA	c.1624G>A	p.E542K	586	207	0.3532
P-0033315-T04-XS1	7	55242469	TTAAGAGAAGCAACATCTC	T	EGFR	c.2240_2257delTAAGAGAAGCAACATCTC	p.L747_P753delinsS	6121	2944	0.481
P-0032790-T04-XS1	12	25398285	C	A	KRAS	c.34G>T	p.G12C	3328	1309	0.3933
P-0048761-T02-XS1	13	32911442	GA	G	BRCA2	c.2957delA	p.N986Ifs*5	4013	1699	0.4234
P-0048761-T02-XS1	13	32911794	A	ATAATT	BRCA2	c.3306_3310dupTTTAA	p.T1104Ifs*2	4464	1580	0.3539
P-0048761-T02-XS1	13	32912428	TTAC	TG	BRCA2	c.3937_3939delinsG	p.Y1313Efs*5	3662	1555	0.4246
P-0048761-T02-XS1	13	32912345	GAA	G	BRCA2	c.3859_3860delAA	p.N1287*	3785	1367	0.3612
P-0050679-T01-XS1	12	25398285	C	A	KRAS	c.34G>T	p.G12C	3078	1428	0.4639
P-0050933-T02-XS1	12	25398285	C	A	KRAS	c.34G>T	p.G12C	7896	4692	0.5942
P-0051884-T01-XS1	6	152419926	A	G	ESR1	c.1613A>G	p.D538G	2087	918	0.4399
P-0051884-T01-XS1	3	178952085	A	G	PIK3CA	c.3140A>G	p.H1047R	2339	1254	0.5361
P-0052299-T01-XS1	14	105246551	C	T	AKT1	c.49G>A	p.E17K	3531	2293	0.6494

Supplementary Table 3. Clinically actionable somatic mutations likely to be removed by standard VAF-based filtering of germline variants.
