# Supplementary material for: Enhanced specificity of clinical high-sensitivity tumor mutation profiling in cell-free DNA via paired normal sequencing using MSK-ACCESS
Source: Nat Commun. 2021 Jun 18;12:3770. doi: 10.1038/s41467-021-24109-5 (PMC8213710; doi:10.1038/s41467-021-24109-5)
Supplement: Supplementary file 3 — Descriptions of Additional Supplementary Files [file 41467_2021_24109_MOESM3_ESM.pdf]

Descriptions of Additional Supplementary Files

Supplementary Data 1
Description: MSK-ACCESS accuracy study results in 70 cfDNA samples with known mutations.

Supplementary Data 2
Description: MSK-ACCESS reproducibility study results.

Supplementary Data 3
Description: Assessment of Limit of Detection (LOD) of the MSK-ACCESS assay.

Supplementary Data 4
Description: Specificity study of the MSK-ACCESS assay (T: true; F: false; N: negative; P: positive).
