# Supplementary material for: Enhanced specificity of clinical high-sensitivity tumor mutation profiling in cell-free DNA via paired normal sequencing using MSK-ACCESS
Source: Nat Commun. 2021 Jun 18;12:3770. doi: 10.1038/s41467-021-24109-5 (PMC8213710; doi:10.1038/s41467-021-24109-5)
Supplement: Supplementary file 8 — Reporting Summary [file 41467_2021_24109_MOESM8_ESM.pdf]

nature research

Corresponding author(s): Ahmet Zehir, Ryma Benayed

Last updated by author(s): Apr 7, 2021

Reporting Summary

Nature Research wishes to improve the reproducibility of the work that we publish. This form provides structure for consistency and transparency in reporting. For further information on Nature Research policies, see our Editorial Policies and the Editorial Policy Checklist.

Statistics

For all statistical analyses, confirm that the following items are present in the figure legend, table legend, main text, or Methods section.

n/a	Confirmed	
☐	☒	The exact sample size (n) for each experimental group/condition, given as a discrete number and unit of measurement
☐	☒	A statement on whether measurements were taken from distinct samples or whether the same sample was measured repeatedly
☐	☒	The statistical test(s) used AND whether they are one- or two-sided<br>*Only common tests should be described solely by name; describe more complex techniques in the Methods section.*
☒	☐	A description of all covariates tested
☒	☐	A description of any assumptions or corrections, such as tests of normality and adjustment for multiple comparisons
☐	☒	A full description of the statistical parameters including central tendency (e.g. means) or other basic estimates (e.g. regression coefficient) AND variation (e.g. standard deviation) or associated estimates of uncertainty (e.g. confidence intervals)
☐	☒	For null hypothesis testing, the test statistic (e.g. F, t, r) with confidence intervals, effect sizes, degrees of freedom and P value noted<br>*Give P values as exact values whenever suitable.*
☒	☐	For Bayesian analysis, information on the choice of priors and Markov chain Monte Carlo settings
☒	☐	For hierarchical and complex designs, identification of the appropriate level for tests and full reporting of outcomes
☐	☒	Estimates of effect sizes (e.g. Cohen's d, Pearson's r), indicating how they were calculated

Our web collection on statistics for biologists contains articles on many of the points above.

Software and code

Policy information about availability of computer code

Data collection	.bam files were generated by BWA MEM and Marianas. MuTect and Vardict were used to generate mutation calls.
Data analysis	Pipeline tools: Trim Galore (v0.2.5), BWA-Mem (v0.7.5a), Marianas (https://github.com/mskcc/Marianas), Waltz (https://github.com/mskcc/Waltz), ABRA (v2.17), GATK (v3.3), Picard (v2.8.1), MuTect (v1.1.5), Vardict(v.1.5.1), GBCM v1.2.2 (https://github.com/mskcc/GetBaseCountsMultiSample), MANTA (v.1.5.0), FACETS v0.5.14, pysam (https://github.com/pysam-developers/pysam) R version 3.6.0.

For manuscripts utilizing custom algorithms or software that are central to the research but not yet described in published literature, software must be made available to editors and reviewers. We strongly encourage code deposition in a community repository (e.g. GitHub). See the Nature Research guidelines for submitting code & software for further information.

Data

Policy information about availability of data

All manuscripts must include a data availability statement. This statement should provide the following information, where applicable:

- Accession codes, unique identifiers, or web links for publicly available datasets
- A list of figures that have associated raw data
- A description of any restrictions on data availability

The mutation data are available to the public through a study on cBio Portal for Cancer Genomics (https://cbioportal.mskcc.org/study/summary?id=msk_access_2021)

Field-specific reporting

Please select the one below that is the best fit for your research. If you are not sure, read the appropriate sections before making your selection.

- [x] Life sciences
- [ ] Behavioural & social sciences
- [ ] Ecological, evolutionary & environmental sciences

For a reference copy of the document with all sections, see nature.com/documents/nr-reporting-summary-flat.pdf

Life sciences study design

All studies must disclose on these points even when the disclosure is negative.

Sample size	Tumor genomic data from first 617 cancer patients tested with MSK-ACCESS was used. We did not perform sample size determination as all patients sequenced were included in the study
Data exclusions	No samples were excluded
Replication	Replication was only performed during the validation of MSK-ACCESS assay. Each patient sample is tested once so no replication was performed for clinical analyses.
Randomization	This is not relevant to our study.
Blinding	The analysis were performed unblinded since we compared results from MSK-IMPACT to MSK-ACCESS

Reporting for specific materials, systems and methods

We require information from authors about some types of materials, experimental systems and methods used in many studies. Here, indicate whether each material, system or method listed is relevant to your study. If you are not sure if a list item applies to your research, read the appropriate section before selecting a response.

Materials & experimental systems

n/a	Involved in the study	
x		Antibodies
x		Eukaryotic cell lines
x		Palaeontology and archaeology
x		Animals and other organisms
	x	Human research participants
	x	Clinical data
x		Dual use research of concern

Methods

n/a	Involved in the study	
x		ChIP-seq
x		Flow cytometry
x		MRI-based neuroimaging

Human research participants

Policy information about studies involving human research participants

Population characteristics	From June 2019 to January 2020, patients with solid tumors seen at Memorial Sloan Kettering Cancer Center (MSKCC) were offered matched tumor-normal DNA sequencing using MSK-ACCESS. No population characteristics covariates are relevant to this study
Recruitment	617 patients were prospectively sequenced in our clinical laboratory following a clinical order made by the treating physician.
Ethics oversight	This study was approved by the MSKCC Institutional Review Board/Privacy Board.

Note that full information on the approval of the study protocol must also be provided in the manuscript.

Clinical data

Policy information about clinical studies

All manuscripts should comply with the ICMJEguidelines for publication of clinical research and a completedCONSORT checklist must be included with all submissions.

Clinical trial registration	NCT01775072
Study protocol	https://clinicaltrials.gov/ct2/show/NCT01775072
Data collection	Tissue and plasma samples were collected from patients who were recruited into the study and MSK-IMPACT and MSK-ACCESS were performed where applicable.

Outcomes

Genomic profiling results were reported back to the ordering clinician and the patient through clinical reports.
